# Proteomic comparison between different tissue preservation methods for identification of promising biomarkers of urothelial bladder cancer

**DOI:** 10.1038/s41598-021-87003-6

**Published:** 2021-04-07

**Authors:** Alberto Valdés, Athanasios Bitzios, Eszter Kassa, Ganna Shevchenko, Alexander Falk, Per-Uno Malmström, Anca Dragomir, Ulrika Segersten, Sara Bergström Lind

**Affiliations:** 1 Department of Chemistry-BMC, Analytical Chemistry, Uppsala University, Box 599, 751 24 Uppsala, Sweden; 2 Laboratory of Foodomics, Institute of Food Science Research, CIAL, CSIC, Nicolás Cabrera 9, 28049 Madrid, Spain; 3 Department of Surgical Science, Urology, Akademiska Hospital, Uppsala University, 751 85 Uppsala, Sweden; 4 Department of Immunology, Genetics and Pathology, Uppsala University, Uppsala, Sweden; 5 Department of Pathology, Uppsala University Hospital, 751 85 Uppsala, Sweden

**Keywords:** Bladder, Bladder cancer, Analytical biochemistry, Mass spectrometry, Proteomic analysis, Oncology, Urology

## Abstract

Samples in biobanks are generally preserved by formalin-fixation and paraffin-embedding (FFPE) and/or optimal cutting temperature compound (OCT)-embedding and subsequently frozen. Mass spectrometry (MS)-based analysis of these samples is now available via developed protocols, however, the differences in results with respect to preservation methods needs further investigation. Here we use bladder urothelial carcinoma tissue of two different tumor stages (Ta/T1—non-muscle invasive bladder cancer (NMIBC), and T2/T3—muscle invasive bladder cancer (MIBC)) which, upon sampling, were divided and preserved by FFPE and OCT. Samples were parallel processed from the two methods and proteins were analyzed with label-free quantitative MS. Over 700 and 1200 proteins were quantified in FFPE and OCT samples, respectively. Multivariate analysis indicates that the preservation method is the main source of variation, but also tumors of different stages could be differentiated. Proteins involved in mitochondrial function were overrepresented in OCT data but missing in the FFPE data, indicating that these proteins are not well preserved by FFPE. Concordant results for proteins such as HMGCS2 (uniquely quantified in Ta/T1 tumors), and LGALS1, ANXA5 and plastin (upregulated in T2/T3 tumors) were observed in both FFPE and OCT data, which supports the use of MS technology for biobank samples and encourages the further evaluation of these proteins as biomarkers.

## Introduction

Protein signaling is important to investigate in order to understand disease mechanisms. Many countries have a long tradition of collecting tissue specimens during surgery and store them in biobanks for further examinations. There are thus large collections of valuable samples resources of, for example, cancer tissue of different stages that can be used for studying how protein expression changes with the development of tumors in relation to clinical parameters such as progression and survival. For long-term storage, two approaches are commonly used: formalin-fixation and paraffin-embedding (FFPE) and optimal cutting temperature compound (OCT)-embedding and subsequent freezing, of the tissue specimens. Analysis of protein signaling in such specimens has mainly been driven by histological staining approaches, e.g. immunohistochemistry (IHC), where only selected proteins can be monitored.

Mass spectrometry (MS)-based proteomics is today a central life science technique in proteomics^1^. The high-resolution-MS enables identification, quantification, and characterization of proteins via the shotgun method^2^ that it is applied to elucidate biological networks and cellular signaling events. The main advantage in using MS is that many proteins can be monitored and quantified at the same time, and therefore remarkable amounts of biological information can be accessed^3,4^. However, the valuable sample resources of cancer tissue stored in biobanks have been difficult to access with MS due to structural changes of the proteins from formaldehyde fixation and contaminations from embedding material. Both paraffin and the OCT-compound, a mixture of polymers (e.g., polyvinyl alcohol and polyethylene glycol), ionize well in the MS and produce characteristic polymer peaks that dominate the spectra if not properly removed. Current advances in the field uses extensive extraction protocols to remove embedding material, such as filter-aided sample preparation (FASP)^5^. This facilitates implementation of additional washing steps to remove polymer residues that otherwise would decrease digestion efficiency, and heat-induced protein extraction in order to reverse the crosslinking for FFPE samples. Despite these barriers, we and other groups have showed that MS analysis has potential in this field^6–9^ and the evaluation of useful samples collected by routine protocols are for MS investigation is important in order to know their value and potential.

We have presented a first optimized protocol for parallel analysis of FFPE and OCT samples using MS-based shotgun proteomics^6^. The model system that was optimized and evaluated was a unique set of urothelial bladder carcinoma tissue that represented different cancer stages, where samples from each patient were preserved using both methods in parallel. More than 2000 and 3000 proteins were identified from FFPE and OCT samples, respectively, and tumors could be preliminarily clustered in stages based on their global protein expression (n = 3 samples per group). Interestingly, totally different sets of deregulated proteins were observed from FFPE and OCT samples of the same tumor.

In this new study, we have used material that has been long-termed stored for 10–15 years and was not specifically dedicated for MS analysis at the time of collection. This tumor tissue collection is unique due to the simultaneous preservation by both FFPE and OCT, which is otherwise uncommon. This makes the study highly relevant for the utilization of long-term stored material but also challenging. Moreover, we have refined the protocol (including a higher number of washes for removal of preservative polymers before protein extraction and in the digestion) and have exhaustively investigated what type of protein expression information can be accessed from the biobanked samples. The developed method was applied to study the similarities and differences of protein expression profiles of non-muscle invasive bladder cancer (NMIBC) (Ta/T1), and muscle-invasive bladder cancer (MIBC) (T2/T3) tumors, preserved by both FFPE and OCT. The results obtained showed that the main source of variation between the samples is the preservation method, but identified promising biomarkers of urinary bladder cancer disease.

## Results and discussion

Out of the 13 individual tumors from patients diagnosed with urinary bladder cancer, nine could be pairwise analyzed after both FFPE and OCT preservation (Table 1). More than 20 samples were initially considered, but due to the limited tissue amounts left in the blocks and/or the low protein extraction efficiency, some samples were excluded. A higher number of proteins could be quantified in OCT-preserved compared to FFPE-preserved samples for all tumors. On average (and without any filtering criteria), 947 and 1511 proteins were quantified in FFPE and OCT, respectively, for T2/T3 tumors; while for Ta/T1 tumors the corresponding numbers were 890 and 1593 (the full list of proteins can be found in Supplementary Table S1). In all, approximately 40% more proteins were quantified in OCT compared to FFPE samples, which agrees with previous observations^6^. The reasons are that the paraffin is more difficult to remove from the FFPE samples than the water-soluble OCT compound, especially for samples with high paraffin-to-tissue ratio, and that the formalin fixation needs to be reversed to remove protein cross-linking. As previously reported, it is preferable for protein extraction to avoid additional detergents, but the use of urea, as in this work, at high temperature to reverse the cross-linking, can modify the proteins through carbamidomethylations^10^. To identify as many proteins as possible, carbamidomethylation was included in the data base search as variable modification since it was previously shown to increase the number of identified proteins in FFPE samples^6^. Still, proteins could have been randomly modified and can therefore not be matched in the data base search. The yield of protein extraction, no matter the preservation method, can also be influenced by the ischemic time, i.e. time between sample collection and preservation, storage time, and by fixation time for FFPE samples. Ideally, it would be advantageous if sliced material from the biobank were transferred directly to Eppendorf tubes suitable for MS-based sample preparation. In this study, this was the case for FFPE samples, but OCT samples needed to be transferred to new vials, which could introduce potential losses of material.

**Table 1:** Number of quantifiable proteins in tumour samples from different patients preserved using different methods.

Tumour stage	Patient	FFPE	OCT	Overlap^a^	r^b^
T2/T3	1	953	–	–	–
	2	827	1579	712	0.768
	6	–	950	–	–
	7	1109	1598	906	0.788
	9	1166	1691	915	0.805
	10	1073	–	–	–
	11	554	1739	434	0.496
	Mean ± SD	947 ± 228	1511 ± 321		
Ta/T1	12	594	1104	508	0.790
	13	1240	1986	1005	0.816
	14	1045	1265	789	0.673
	15	937	–	–	–
	19	1069	1823	901	0.773
	20	453	1787	379	0.499
	Mean ± SD	890 ± 303	1593 ± 385		

^a^Number of proteins commonly quantified in tumours preserved by different approaches.

^b^The correlations of quantitative measured values, the normalized LFQ intensities, from FFPE and OCT samples, presented by Pearson’s correlation coefficients (r).

### Comparison of preservation methods

**Figure 1:**
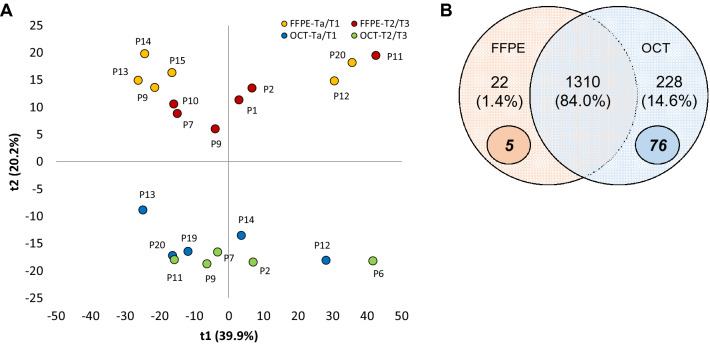
(**A**) PCA score plot (t2 vs t1) of data from proteins quantified in at least three sample replicates of each preservation method (FFPE and OCT) and each tumor stage (Ta/T1 and T2/T3). (**B**) Venn Diagram of overlapped proteins quantified in at least 50% of FFPE and OCT samples (including samples from the two different stages). Unique proteins for each preservation method are marked in bold and italics inside small circles. Created using Microsoft Office Professional Plus 2016.

To get an overview of the variation between all groups (FFPE-Ta/T1, FFPE-T2/T3, OCT-Ta/T1 and OCT-T2/T3), the expression of 656 shared proteins (those proteins simultaneously quantified in at least 50% of the samples in each group) was analyzed by principal component analysis (PCA) (Fig. 1A). The samples mainly clustered based on the preservation method, indicating that the preservation method introduced larger variation than the tumor stage to the sample differences. The main difference is likely due to the different scale of label-free quantification (LFQ) intensities, indicating that if data from different preservations is to be combined, attention must be paid to proper normalization.

Moreover, to investigate the differences between the two preservation methods (without considering the tumor stages), those proteins quantified in at least 50% of the samples (in 6 FFPE samples and/or 5 OCT samples) were considered. Following this filtering criteria, 1560 proteins were quantified. The Venn Diagram (Fig. 1B) shows that 84% of the proteins were quantified in both FFPE and OCT samples, while 228 proteins were mainly quantified in OCT samples, and 22 proteins were mainly quantified in FFPE samples.

**Figure 2:**
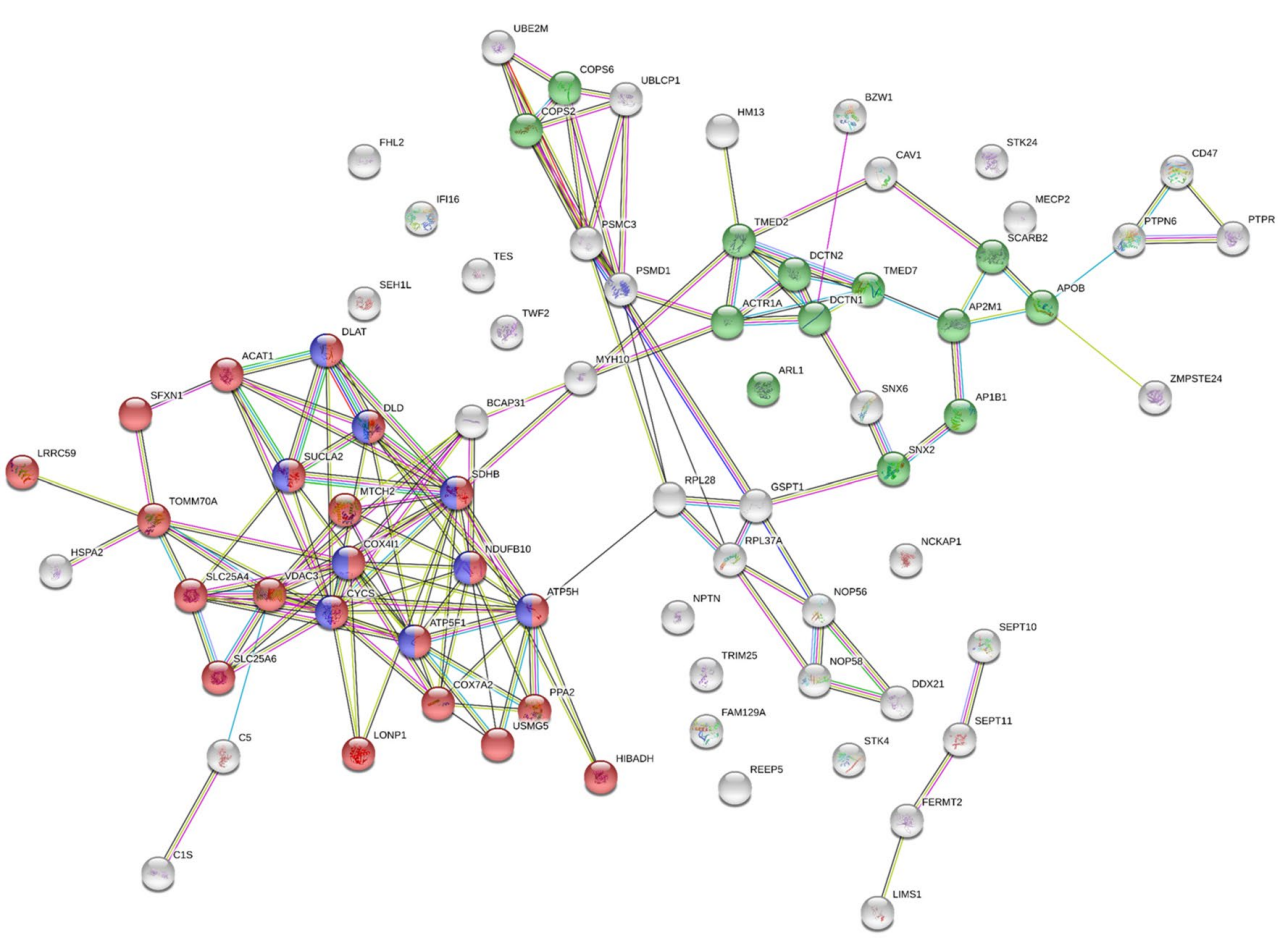
STRING diagram representing protein interaction pathway derived from the 77 proteins quantified in at least 80% of OCT and in none FFPE samples. The average local clustering coefficient as reported by STRING is 0.478. Proteins in blue belong to the *TCA cycle and respiratory electron transport* and proteins in green belong to the *Membrane Trafficking* (Reactome Pathways). Proteins in red belong to the *Mitochondrial part* (Cellular Component GO). Obtained from STRING v11 web-based software (https://string-db.org/).

Among the proteins mainly quantified in one preservation method, 76 and 5 were considered unique (proteins quantified in at least 80% samples of one preservation method and in zero samples in the other method) in OCT and FFPE samples, respectively (Supplementary Table S2). The analysis of the FFPE-unique proteins using STRING software did not show any significant result. However, the *TCA (tricarboxylic acid or the Krebs) cycle and respiratory electron transport* and the *Membrane Trafficking* pathways were the most enriched Reactome’s pathways in OCT unique proteins (Fig. 2). Many of these proteins were also considered in the *Mitochondrial part* of the gene ontology (GO) Cellular Component term. The list of pathways and GO terms enriched in OCT data set can be found in Supplementary Table S3. We propose that these proteins are not as well preserved in FFPE as in OCT, which raise an important and general aspect on how preservation methods are selected in relation to the study aims. The fact that the LFQ-intensities of these proteins were relatively high, and not close to the limit of detection, supports this theory.

### Paired tumor comparison

To investigate how the proteomes of tumors of different stages are affected by the method of preservation, the proteomes of each tumor tissue that had data for the two preservation methods (n = 4 for T2/T3 and n = 5 for Ta/T1) were compared. The number of overlapping proteins was found to be in general slightly less than the number of quantifiable ones in FFPE samples, varying from around 400 to 1000 proteins depending on the tumor. The number of quantifiable proteins in FFPE samples is, as described above, limited compared to OCT samples. The average overlap of proteins observed when comparing the same sample but differently preserved was 41% (Supplementary Fig. S1), which agrees well with our initial study of method development (with an overlap of 45%^6^). These results differ from the results obtained when considering all the samples together (84%, as shown in Fig. 1B), where no distinction between the stages was applied, and highlights the high variability of the quantified proteins coming from the same sample preserved differently. These discrepancies could be explained by the difference in ischemic time and other pre-analytical parameters for the two preservation methods, or due to the heterogeneity of the tumors when sampling for their preservation.

When correlating the quantitative intensities from these overlapping proteins, a trend for higher Pearson’s correlation with higher number of overlapping quantifiable proteins was observed. For a tumor with around 400 quantified proteins, the coefficient correlation was ≈ 0.5, but for a tumor with around 1000 quantified proteins, the correlation coefficient was ≈ 0.8. The low correlation for some tumors could be explained by the heterogeneity of samples, or due to admixing non-tumoral stromal/muscle tissue with a few highly proteins that masked the detection of tumor proteins. Considering all samples, the average correlation was 0.72 (Supplementary Figs. S2 and S3), which is considered rather good for comparative studies of protein expression. This implies that the improved protocols for extracting proteins from tissue preserved by the two methods^6^ provides concordant results for the overlapping proteins, even though the scales of the LFQ intensities of OCT and FFPE preserved samples might differ.

### Comparison of tumor stages

**Figure 3:**
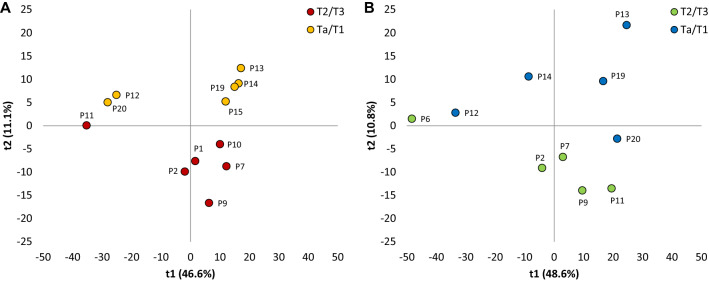
PCA score plots (t2 vs t1) of data from proteins commonly found among samples according to their tumour stages samples preserved by FFPE (**A**) and OCT (**B**). For FFPE: Patients 1, 7, 9, 10 and 11 were diagnosed with stage T2/T3, and patients 12, 13, 14, 15, 19 and 20 were diagnosed with stage Ta/T1. For OCT: Patients 2, 6, 7, 9 and 11 were diagnosed with stage T2/T3, and patients 12, 13, 14, 19 and 20 were diagnosed with stage Ta/T1. Created using Microsoft Office Professional Plus 2016.

Next, FFPE and OCT samples were independently evaluated for biological information of the different tumor stages using PCA. For this analysis, proteins that were quantified in at least 50% of the samples among the two tumor stages categories within each preservation method were used. For FFPE samples (757 proteins), PC1 and PC2 together represented 58% of the variation (Fig. 3A). Different patients, representing a certain stage, could be clustered into the same group, showing that the samples from the same stages were similar to each other. For OCT samples (1225 proteins), PC1 and PC2 represented 59% of the variation (Fig. 3B) and the T2/T3 stage tumors clustered better compared to the Ta/T1 tumors. For the OCT samples, the clustering of tumors into different stages was not as pronounced as for FFPE samples. One possible reason for the different results between FFPE and OCT samples is that slightly different patient’s samples were included in the two PCAs (Table 1), and also a different number of proteins (757 vs 1225). It should be noted that PCA aims for finding the largest source of variation in the data set, without considering groups, and that the absence of a great distinction between the groups does not mean the absence of significant differences between them. In this sense, the higher number of extracted proteins from OCT samples could hinder those proteins that could separate the two groups in the PCA.

Given the separation using the PCA, the supervised partial least squares discriminant analysis (PLS-DA) analysis was applied for sample classification, to estimate the importance of each protein in the separation of the two groups (based on the variable importance in the projection (VIP) values), and to identify those common proteins than could separate the two stages when using the different preservation methods. Based on the “Leave-one-out” cross-validation method, two components were selected for both FFPE (R^2^Y = 0.952, Q^2^ = 0.393) and OCT (R^2^Y = 0.954, Q^2^ = 0.141) preservation methods. The R^2^Y metric describes the percentage of variation explained by the model, and indicates that the variability of both models is well explained, and that the samples are perfectly separated (Supporting Fig. S4). On the other hand, the Q^2^ metric represents the predictive ability of the model, indicating that the predictive ability of the obtained models is rather low. According to these models, 97 and 105 proteins had VIP values > 1.5 in FFPE and OCT samples, respectively. The whole list of proteins, with their VIP and coefficient values can be found in Supplementary Table S4. Interestingly, 18 proteins with a VIP values > 1.5 were common between the FFPE and the OCT samples (**TGFBI, ANXA5, TPT1, FN1, CORO1C, LGALS1, PGM1, MAOA, ETFB, ACTB, FTH1, ANXA1, ACTN1, SERPINH1, CAT, CAP1, ACTN4** and **AKR1C1**). These proteins could be used for the classification of the different stages in both preservation methods, but it has to be taken into account that for the PCA and PLS-DA analyses, some LFQ intensity values were imputed, and therefore univariate analyses (described below) are necessary to validate their importance. Moreover, the low Q^2^ values, the different number of identified proteins in the differently preserved samples, and the variable LFQ intensities obtained between the FFPE and the OCT samples do not allow to create robust and predictive models to be used for the classification of samples no matter which preservation method is used.

Finally, univariate analyses were performed to compare the two tumor stages based on the two sample sets of FFPE and OCT samples independently. The comparable numbers of proteins in the FFPE and OCT sets (Table 2) were approximately 20% lower than the average of quantifiable proteins per sample (Table 1). As presented in Table 2 and in the Volcano plots presented in Supplementary Fig. S5, a higher number of proteins were obtained in the OCT compared to the FFPE sample set. The full lists of differentially expressed and unique proteins in FFPE and OCT samples are presented in Supplementary Tables S5–S8.

**Table 2:** Number of proteins obtained from different samples.

Parameter	FFPE	OCT
Quantified proteins (in three or more samples)	757	1225
Proteins (p < 0.05) (T2/T3 vs Ta/T1)	83	92
Up-regulated proteins (T2/T3 vs Ta/T1)^a^	13	16
Down-regulated proteins (T2/T3 vs Ta/T1)^b^	21	32
Unique proteins (T2/T3)	1	7
Unique proteins (Ta/T1)	1	2

^a^Log2 Fold change ≥ 1 and p-value ≤ 0.05.

^b^Log2 Fold change ≤ − 1 and p-value ≤ 0.05.

#### Interesting differentially expressed proteins overlapping in FFPE and OCT data sets

In order to find biomarkers that can distinguish between different cancer stages, the differentially expressed proteins and the unique proteins were investigated. Table 3 summarizes proteins that were commonly quantified with a p < 0.05 between tumor stages in both data sets with fold change > 2 in FFPE or/and OCT tumors, and their relevance in cancer research. All proteins overlapping in this list show the same trend of regulation, even though not all proteins are significantly altered in both FFPE and OCT samples. To pinpoint the most striking results:

**Table 3:** Differentially expressed proteins and unique proteins commonly quantified in FFPE and OCT preserved samples (T2/T3 vs Ta/T1) and their relevance in cancer research.

Protein ID	Protein name	Gene name	FFPE (T2/T3 vs Ta/T1)		OCT (T2/T3 vs Ta/T1)		Cancer relevance	Ref
			Log2 fold change	P-value	Log2 fold change	P-value		
P09382	Galectin-1	LGALS1	**2.18**	0.019	**1.42**	0.004	Angiogenesis, invasion, cell–cell and cell–matrix interactions important for metastasis	^11,37^
P08758	Annexin A5	ANXA5	**1.39**	0.001	0.95	0.001	Tumor progression, invasion, metastasis, drug resistance	^12,13^
P31930	Cytochrome b-c1 complex subunit 1, mitochondrial	UQCRC1	− 0.73	0.001	*− 1.02*	0.017	Highly expressed in breast and ovarian tumors	^38^
Q96I99	Succinyl-CoA ligase [GDP-forming] subunit beta, mitochondrial	SUCLG2	− 0.77	0.034	*− 1.57*	0.030	Decreased expression in renal carcinoma	^39^
P38117	Electron transfer flavoprotein subunit beta	ETFB	− 0.81	0.026	*− 1.43*	0.017	Biomarker of follicular carcinoma	^40^
P39687	Acidic leucine-rich nuclear phosphoprotein 32 family member A	ANP32A	− 0.92	0.035	*− 1.03*	0.017	Promotes colorectal cancer proliferation	^41^
P51532	Transcription activator BRG1	SMARCA4	*− 1.37*	0.010	− 0.71	0.038	Tumor suppressor	^42^
P49411	Elongation factor Tu, mitochondrial	TUFM	*− 1.20*	0.039	*− 1.03*	0.035	Bad prognosis in colorectal and lung cancer	^14,15^
P54868	Hydroxymethylglutaryl-CoA synthase, mitochondrial	HMGCS2	*Unique in Ta/T1*		*Unique in Ta/T1*		Prognosis biomarker in several cancer tissues	^16–19^

Deregulated proteins are emphasised (bold—upregulated, italics—downregulated).

**Figure 4:**
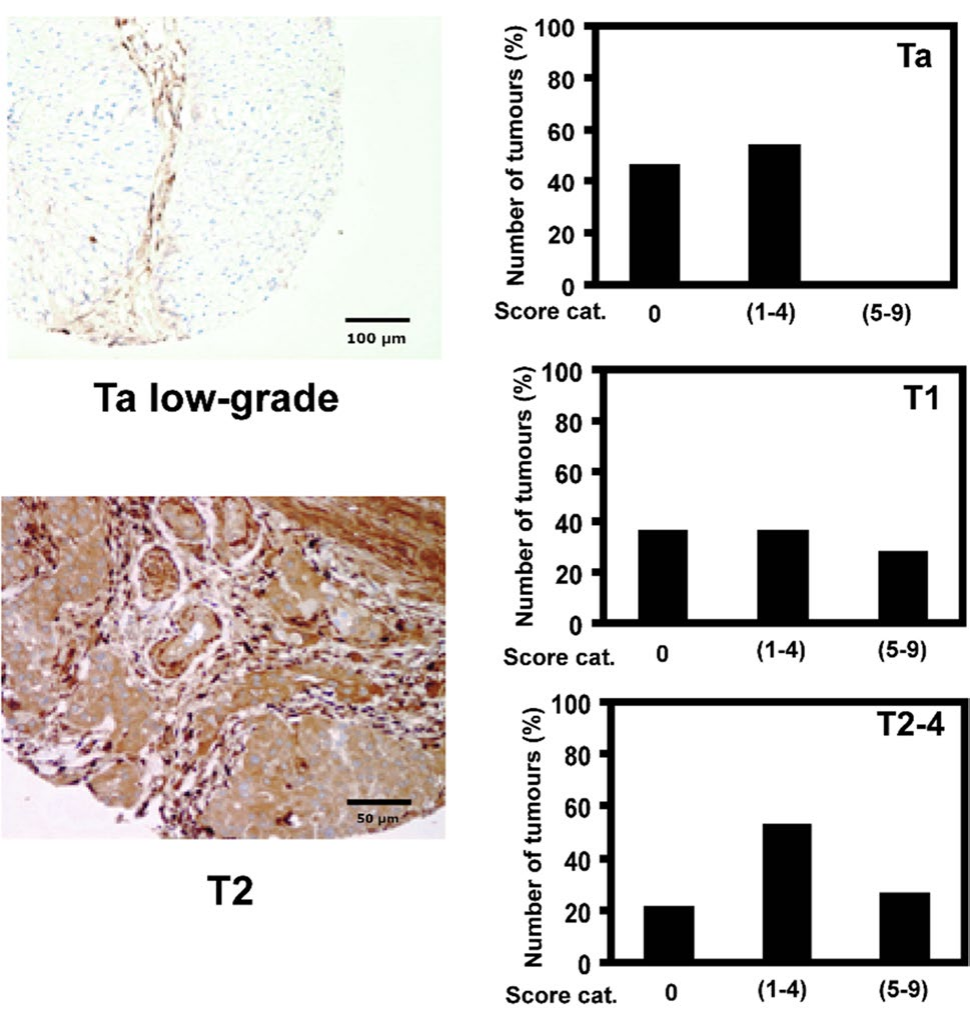
Expression of GAL1 in bladder cancer: Left, IHC images. Scale is specified in the panels. Right, score distribution between stages (Ta, T1 and T2–4). This analysis showed that the cytoplasmic GAL1 expression in the tumor cells was significantly higher (P < 0.05) in T1 tumors and in poorly differentiated stages (T2–4) than in Ta tumors. Stroma and blood vessels also stained positively in all tumors. Only the tumor cell‐staining was scored. Reproduced with modifications from^11^, with permission from BJU International, John Wiley & Sons.

Galectin-1 (LGALS1 or GAL1) was clearly upregulated in T2/T3 tumors and this finding agreed for both preservation methods. To support the MS-based results, our previous study that screened the publicly available Human protein atlas (HPA 1.0) for immunohistochemistry staining patterns representative for urinary bladder cancer^11^ was consulted. The GAL1 protein was one of the proteins that indicated differential expression in bladder cancer. The protein was therefore further evaluated using tissue micro array and the results showed that the GAL1 protein expression was significantly increased in tumors of high stage compared to low stage tumors. The results are presented in Fig. 4, (reproduced from BJUI with permission).

Annexin A5 (ANXA5) was also upregulated in T2/T3 tumors, which agrees well with what has already been reported in literature^12^. For bladder cancer specifically, this protein has been suggested to be a marker of the transition from low stage to high stage tumors using the tissue microarray technology^13^. In our dataset, ANXA5 had more than a two-fold upregulation in the FFPE T2/T3 versus Ta/T1 sample set and was almost two-fold upregulated in OCT samples for the same comparison of tumors.

Elongation factor Tu, mitochondrial (TUFM) was down-regulated in T2/T3 tumors (implies higher expression in Ta/T1 tumors). The TUFM protein has been related with bad prognosis in colorectal^14^ and lung cancer^15^ but no previous reports of associating the TUFM protein to urinary bladder cancer have, to the authors’ knowledge, been reported.

Among regulated proteins, unique proteins can be regarded as the most upregulated in respective group, even though fold change values cannot be calculated since they were not quantified in the other group. The HMGC2 (Hydroxymethylglutaryl-CoA synthase, mitochondrial protein) was uniquely observed in Ta/T1 tumors in both FFPE and OCT samples (Table 3) which makes it a strong candidate as a protein that is highly involved in low stage tumors. The HMGCS2 protein has been related with tumor initiation or metastasis^16^, or acting as a tumor suppressor^17^. This protein has been reported as decreased in different cancer tissues (prostate, colon, liver), and it has been suggested as a prognosis biomarker^18,19^. According to the authors’ knowledge, this protein has not been reported associated with urinary bladder cancer before, but due to the observations of unique expression in low cancer stages presented here, verification, e.g. using large cohorts of collected tumor tissue specimens with correlated clinical data, will be of interest in the future.

Another interesting group of proteins are Plastins. Plastin 2 (LCP1, log twofold change 1.43) was identified as the 10th most upregulated protein in FFPE samples, while Plastin 3 (PLS3, log twofold change 1.41) was one of the top-upregulated proteins in OCT samples (Supplementary Tables S5 and S6). These proteins have 80% similarity in sequence and have been reported to have similar functions. Both proteins are involved in actin binding. In urinary bladder cancer, LPC1 has previously been reported upregulated in high stages tumors by TMA^13^ and by IHC staining^20^.

#### Interesting differentially expressed proteins non-overlapping in FFPE and OCT data sets

Besides HMGC2, the **PAICS** (Multifunctional protein ADE2/phosphoribosyl aminoimidazole succinocarboxamide synthetase) protein was uniquely observed in T2/T3 tumors in FFPE samples. (Supplementary Table S7). In OCT samples, the **TST** (Thiosulfate sulfur transferase) protein was uniquely observed in Ta/T1 tumors (Supplementary Table S8). The **SEMG1** (Semenogelin-1), **CYBB** (Cytochrome b-245 heavy chain), **AK4** (Adenylate kinase 4, mitochondrial), **PDLIM4** (PDZ and LIM domain protein 4), **PCOLCE** (Procollagen C-endopeptidase enhancer 1), **AHNAK2** (Protein AHNAK2) and **MYADM** (Myeloid-associated differentiation marker) in T2/T3 tumors of OCT preservation (Supplementary Table S8). Of these proteins, only three have been previously discussed in bladder cancer; **PAICS** has previously been identified as overexpressed in this type of cancer^21^, increased **AK4** expression has been correlated with poor prognosis and involved in progression of disease^21^ and **AHNAK2** has been reported as a marker that can differentiate between inflammation with reactive urothelial atypia and carcinoma in situ^22^. The other proteins are according to our observation not known to be previously related to bladder cancer and are potential new molecular markers to be subjected to follow-up studies.

More biomarker candidates were the top-upregulated proteins in T2/T3 tumors compared to Ta/T1 tumors from the FFPE set were **TGFBI, FN1, FTL, CD44, G6PD** and **CLU** and the most downregulated proteins in the same comparison were **NUMA1, AKR1C1, VARS, CTTNNA1** and **PRKDC**. In the same way, the most upregulated proteins in T2/T3 tumors compared to Ta/T1 in the OCT data set were **DSP, ANXA1, CORO1C, TUBB6** and **TES** and the correspondingly downregulated proteins were **SLC4A1, HBD, SSH3, CRABP2** and **HBB**. Many of these proteins were already reported in the PLS-DA analysis to be proteins that separate the tumor groups (Supplementary Table S4) while others, such as the well-known and reviewed breast cancer marker CD44^23^, was only observed regulated in this univariate analysis. HBB and HBD are related to hemoglobin and should most likely be regarded as possible contaminants in this analysis.

#### Comparison with previously published protein and RNA data sets

The lists of differentially expressed proteins were compared to a recently published work focused on the classification of NMIBC by MS-based proteomics by Striggilos et al.^24^. In that work, the authors used fresh frozen tissues from 98 NMIBC patients and 19 MIBC patients, at primary diagnosis. Based on their proteomics results, the NMIBC patients could be stratified into three subtypes: NPS1 (mostly high stage/grade/risk samples), NPS2 (mixed stage/grade/risk composition) and NPS3 (mostly low stage/grade/risk samples). In addition, the authors showed that NPS1 samples were closed to MIBC, and quite different from NPS2 and NPS3. Due to the limited number of paired tumor samples simultaneously preserved by OCT and FFPE in the present study, the tumor samples were pooled as Ta/T1 and T2/T3 to increase the statistical power, being aware of the possible heterogeneity of each group from a molecular perspective^18,19,24^. In contrast to Stroggilos et al.^24^, a fold change cut-off filter was applied to increase the confidence in the identification of significantly altered proteins between Ta/T1 and T2/T3 groups. Besides these differences, the expression of some significantly altered proteins agreed well in both studies (**G6PD**, **COMT**, **TYMP** and **ABHD14** in FFPE preserved samples; **SSH3**, **CYB5R1** and **METT7A** in OCT preserved samples). This manuscript identified several altered proteins in one specific group of samples (MIBC/NPS1/NPS2/NPS3). Among them, **TGFBI**, **BGN**, **DPYSL2** and **TUBB** had a very low expression in NPS3, which might agree with the up-regulation in T2/T3 samples observed in our study; **PAICS** was mainly observed in NPS1, and it was identified as unique in T2/T3 samples; and **CTNNA1** was increased in NPS3, and it was significantly decreased in T2/T3 samples.

The significantly altered proteins in our study were also compared to RNA data sets from different urothelial carcinoma tissues that are deposited at the Gene Expression Omnibus public repository (GSE32894^19^; GSE83586^18^). The data were processed using the GEO2R interactive web tool and the following groups of tumors were defined: in GSE32894 data set, 213 NMI samples (Ta/T1) were compared against 93 MI samples (T2/T3/T4); and in GSE83586 data set, 58 NMI samples (Ta/T1/Tis) were compared against 243 MI samples (T2/T3/T4). In overall, the agreement between our proteomic data and the two RNA data sets is good (see Supplementary Table S9 and Table S10), which strengthens the results obtained in the actual proteomics work. As an example, the HMGCS gene was observed as one of the most down-regulated genes in MIBC from GSE32894, and it was significantly down-regulated in MIBC from GSE83586, which agrees with our finding as unique in Ta/T1 group.

Combining the results from the FFPE and OCT datasets from our initial study of method development^6^ and the present investigation, eight proteins were common and differentially expressed in the same direction (Supplementary Table S11). **LCP1** (Plastin-2), **ANXA5**, **DPYSL2** (dihydripyramidinase-related protein 2) and **BGN** (Biglycan) were upregulated in T2/T3 tumors compared to Ta/T1 tumors in both studies. Downregulated in both studies were **HSD17B4** (peroxisomal multifunctional enzyme type 2), **UQCRC1** (Cytochrome b-c1 complex subunit 1, mitochondrial) and **VARS** (Valine-tRNA ligase). Notably, the deregulation of these proteins were all observed in FFPE samples from previous study, but could also be confirmed by either FFPE or OCT data in the present study. Since no differentially expressed proteins from OCT preserved samples overlapped between this and the previous study (Supplementary Table S11) and due to the unambiguous results between OCT and FFPE data sets observed in this study, we conclude that the initial study from the OCT screening of only three tumors was not big enough to generate reliable data.

## General overview

As the number of reports relying on biobanked data is increasing, we need information on how to make a reliably study. Comparing proteins identified in samples from one and the same preservation method most likely produce reliable results, provided standardized preservation and sampling handling thereafter. Table 3 points out that just a few differentially expressed proteins between the two tumor stages overlapped in the two sample sets of FFPE and OCT. This result is not surprising since no overlap at all was observed in our initial study^6^. This study shows encouraging but not complete unanimous results. Notably, six of the deregulated proteins in OCT and FFPE datasets, actually overlapped with the uniquely quantified proteins when comparing OCT and FFPE data. Two examples are the **TES** (Testin) protein and **DDX21** (Nucleolar RNA helicase 2) that were among the top five up-regulated protein in OCT data but was not even quantified in FFPE samples. These proteins are likely better preserved or detected from OCT approach compared to FFPE approach. The low overlap of deregulated proteins can be due to several reasons; (1) the tumor groups for FFPE and OCT sets were not identical, even though most tumors originated from the same patient origin for FFPE and OCT sample sets (Table 1); (2) the preservation processes of FFPE and OCT are different and some proteins are better preserved by either way; (3) the procedures for handling the sample sets are slightly different, even though as many steps as possible are performed in parallel. This could imply that some groups of proteins could be damaged or selectively eliminated in the procedures to remove the embedding material; (4) fewer proteins in general were identified, quantified and deregulated in FFPE samples compared to OCT samples. In a larger perspective, it is a concern that the results of proteomic studies of preserved tissue to identify e.g. biomarkers of disease might need complementary analyses of both FFPE and OCT material to fully cover all angles. There are only a few reports on the proteome comparison of FFPE and other types of preserved tissue using MS analysis. One study compared snap frozen tissue with FFPE where the overlap of quantified proteins was > 90%^25^ and another study that compared OCT and FFPE, even though with different specimens, reports on reduced protein identification in FFPE samples compared to OCT samples^26^. In such comparisons, the heterogeneity of tumors needs to be concerned. Not exactly the same piece of a tumor can be preserved with different approaches. In this study we have taken this into account by manual staging of the preserved tumors by a trained pathologist, and only tumor with the same classification have been concerned. Our comparison of proteomes is unique due to the parallel preservation and processing of one and the same tumor specimens, and adds therefore important and valuable information.

## Conclusion

By using MS-based proteomics for analysis of tissue stored in biobanks, such as FFPE and OCT embedded and subsequently frozen samples, we show that important proteins that have potential of being biomarkers in cancer can be identified, quantified and compared between different groups. Provided appropriate sample preparation, the analysis affords comparison of a high number of proteins, and due to the unique collection of samples where tumors have been preserved with both approaches, we can draw important conclusions. In the whole data set, the largest difference lies in the method of preservation, that in turn can probably be due to variable levels of protein intensities observed in MS in the two methods, implying that samples and/or datasets from different preservations should not be combined. Generally, a higher number of proteins could be analyzed in the OCT than in the FFPE data sets. The OCT data was, however, more dispersed and the FFPE data had better and more consistent clustering of samples when comparing different tumor stages. The deregulated proteins did not fully overlap between different tumor stages in OCT and FFPE, but nine cancer-related proteins were changed in the same way in both methods. Among them, the Galectin-1 protein, upregulated in T2/T3 tumors, was previously validated in an independent material, where it showed a significantly higher cytoplasmic IHC staining in tumors of high stage compared to tumors of low stage. Interestingly, other cancer-relevant proteins were observed as deregulated between different tumor stages in unique data sets. The fact that proteins that could not be quantified in FFPE but in OCT samples are related to mitochondrial function is an important aspect, indicating that such proteins require attention in comparative studies using samples from biobanks. In a general perspective, complementary analysis of different type of preserved tissue might be needed to cover all angles of proteomics investigation.

## Methods

### Sample cohort for MS analysis

The sample cohort consisted of tissue samples from urinary bladder cancer patients diagnosed with urothelial carcinoma at non-muscle invasive stage (Ta/T1) or muscle invasive stage (T2/T3). The samples were collected after transurethral surgical resection at Uppsala University Hospital (Sweden) between 2005 and 2009. Ethical permission for the investigations was granted by Uppsala University (DNR 2015-143-1). Informed consent for study participation is attested. All methods were performed in accordance with the relevant guidelines and regulations. Specimens from 13 individual patients were bisected and preserved (see below) within 1–2 h after surgery by either FFPE or OCT/freezing approaches. Tumors were staged according to the tumor node metastasis (TNM)^27^ system by an expert pathologist. Tumors from 20 patients diagnosed with urinary bladder cancer were considered, but narrowed down to 13 tumors from individual patients that were subjected to analysis. Nine of these could be investigated in pairs with both FFPE-OCT (see specifications in “Results and discussion” section).

### Tumor tissue preservation

For the OCT/frozen method, samples were incubated with Tissue-Tek O.C.T. compound (Sakura, The Netherlands) (ischemic time 1–4 h) and snap frozen in liquid nitrogen. The tissue blocks were sectioned into 10 µm slices using a cryostat at ≤ − 20 °C. Five consecutive sections were pooled and used as one sample for MS investigation and stored at − 80 °C until analysis.

The FFPE method included an initial step of standardized fixation of the tissue in 4% buffered formaldehyde solution (ischemic time < 5 min) for approximately 24 h after which the tissue was dehydrated and embedded in paraffin as per clinical routines. The paraffin blocks were sectioned into 10 µm slices. Five consecutive sections were pooled in Eppendorf tubes and used as one sample for MS analysis and stored at room temperature (RT) until use.

### Deparaffinization and rehydration of FFPE

The paraffin embedded tissue processed as previously described^6^ with minor modifications in the number of xylene washes. Briefly, paraffin was removed from tissue slices by five washes of xylene and one incubation with heptane that was complemented with an addition of methanol at the end. After removing the upper heptane layer, the remaining methanol was evaporated before sequential rehydration; a series of ethanol washes (100% (v/v; two times)), 90%, 80%, 70% and a final wash with Milli-Q-water was applied.

### OCT removal

The OCT embedded tissue was processed as previously described^6^ with minor changes regarding the number of washing steps. Briefly, the tissue sections were washed with ethanol (70%) five times, four times with water, and three times with 50 mM ammonium bicarbonate. All washing solutions were freshly prepared and ice-cold. Prior to further processing the final washing solution was removed and the tissue was left to air-dry at RT for 5 min.

### Protein extraction

OCT and FFPE preserved tissue samples were treated in parallel as described by Holfeld et al.^6^. Briefly, a lysis buffer consisting of 7 M urea, 2 M thiourea, 1 M ammoniumbicarbonate, protease and phosphatase inhibitor cocktails was added to the cleared-out tissue specimens, followed in the case of FFPE tissue, by heating to 95 °C for 30 min and incubation at 60 °C for 2 h in order to reverse the cross linking was applied. This was followed by routine procedures of sonication and centrifugation for both FFPE and OCT samples.

### Protein digestion

Multi-enzyme digestion using filter aided sample preparation, originally developed by Wisniewski et al.^28^ but modified by Holfeld et al.^6^, was used. Concisely, total protein concentrations were determined using Bio-Rad protein assay (Bio-Rad, Hercules, California, U.S.A) and normalized amounts of 10 µg total protein were selected and digested, after reduction and alkylation, on molecular weight cut off filters (Amicon Ultra 10 K, Merck Millipore, Burlington, MA, U.S.A.) with a combination of Lys C and trypsin. The resulting peptides were collected by centrifugation of the device and collecting the generated flow through. Peptides were acidified with addition of trifluoroacetic acid (TFA) and cleaned up using Pierce C18 Spin Columns (Thermo Fisher Scientific, Bremen, Germany).

### Nano liquid chromatography tandem mass spectrometry (LC–MS/MS)

LC–MS/MS analysis was performed via an EASY-nLC 1000 system (Thermo Fisher Scientific) coupled to an QExactive Plus mass spectrometer (Thermo Fischer Scientific) equipped with a nano-ESI source. Peptides were reconstituted in 30 µL 0.1% formic acid (FA) of which 5 µL was loaded onto a pre-column (2 cm long nano viper (NV)-column, 3 µm particle size, C18; Thermo Fischer Scientific). Peptide separation was carried out using a linear 90 min gradient from 4 to 100% B (ACN, 0.1% FA) with a flow rate of 250 nL/min through the pre-column and the following analytical column (NV column ID 75 µM, 2 µm particle size, 15 cm long, C18; Thermo Fischer Scientific). The mass spectrometer operated in positive ion mode with an electrospray voltage of 2–2.2 kV. The survey MS spectra (400–1750 m*/z* range) were generated with a resolving power of 70,000 (full width half-maximum, full width half maximum) using an automatic gain control (AGC) target of 3 × 10^6^. Data was recorded in data-dependent mode where the top ten most abundant peaks were selected for fragmentation using higher energy collision-induced fragmentation. A collision energy of 25% was applied. An AGC target for MS/MS was set to 5 × 10^5^ at a resolution of 17,500. Dynamic exclusion was used with an exclusion period of 20 s.

### Data analysis

Acquired RAW MS files were processed using MaxQuant software (version 1.5.7.4)^29^ with the integrated Andromeda search engine^30^. The spectra were matched against the UniProt human database (Homo sapiens, downloaded on July 2019, containing 26,483 proteins sequences). A target-decoy search, using a reversed database, was used to calculate the false discovery rate (FDR) and 1% FDR was accepted for protein and peptide identification. The following settings were used in the search: minimum peptide length of seven amino acids, 20 ppm mass tolerance in first search was, 4.5 ppm mass tolerance in the second search, 20 ppm mass tolerance for the fragment ions, trypsin as digestive enzyme and two maximum missed cleavages were allowed. For all searches, carbamidomehtylation of cysteine residues was set as fixed modification, and oxidation of methionine and N-carbamylation were included as variable modification. For protein quantification, LFQ intensities were used and only proteins with at least two identified peptides were included. The mass spectrometry proteomics data have been deposited to the ProteomeXchange Consortium^31^ via the PRIDE^32^ partner repository with the dataset identifier PXD020194.

### Statistical analysis

For statistical analysis, MaxQuant “proteingroups.txt” files were loaded into Perseus software (version 1.6.2.3)^33^. Potential contaminants and reverse hits were removed from the protein list and LFQ intensities were log2 transformed to test their normal distribution using histograms. PCA of proteins quantified in at least three replicates of each group (FFPE-Ta/T1, FFPE-T2/T3, OCT-Ta/T1 and OCT-T2/T3) was carried out using Statistica software (version 13.3, TIBCO Software, Inc., CA, U.S.A) to visualize the projection of the data sets. Missing values were replaced by random numbers that are drawn from a normal distribution and data were auto-scaled. Unique proteins between the different preservation methods were defined as proteins quantified in at least 80% samples of one preservation method and in zero samples in the other method. Moreover, unique proteins for the different stages within the same preservation method were defined as proteins quantified in at least 80% samples of one tumor stage category and in zero samples in the other tumor stage category. The lists of unique proteins were then searched for enriched Reactome or Kegg pathways, or GO terms using STRING v11 web-based software^34^. Pathways or GO terms were considered significant when p-value < 0.05 after Benjamini and Hochberg FDR correction. In those cases, where the same tumor tissue was preserved by both FFPE and OCT methods, log2 LFQ intensities of the quantified proteins were plotted against each other, and the correlation was evaluated using the Pearson’s correlation coefficient.

Samples from the same preservation method were analyzed independently. PCA and PLS-DA of proteins commonly quantified in at least 50% of the samples among the two tumor stages within each preservation method were analyzed using Statistica software. VIP values of the selected components of the PLS models were used to select important proteins. Finally, univariate Student T test analysis was applied for proteins that were quantified in at least 50% of the samples per group to determine differentially expressed proteins between the two different stages. Proteins were considering significantly expressed when p < 0.05 and log2 fold change ≤ − 1 or ≥ 1. Data were visualized using Volcano plots generated by plotting the log2 fold changes against the negative logarithm of the p-values.

### Immunohistochemistry validation

Findings of upregulated LGALS1 (also GAL-1) in high stages tumors compared to low stages tumors were validated using IHC of antibodies from the Human Protein Atlas^35^. The validation is part of a previously published paper by Segersten et al.^11^ that gives the experimental details. Briefly, the HPA version 1.0 was used to screen for proteins that showed characteristic staining by IHC in urinary bladder cancer. Thereafter, selected proteins such as GAL1 were evaluated using IHC on a tissue micro array TMA, consisting of independent bladder cancer tumor material classified according to the WHO grading system^36^. The study was approved by the regional ethical review board of Uppsala (reference number 2005: 339) and comprised 29 Ta (seven papillary urothelial neoplasm of low malignant potential, 10 low-grade and 12 high-grade), 34 T1 (six low-grade and 28 high-grade), 43 T2–T4 (all high-grade) tumours and five morphologically normal urothelium samples. All methods were performed in accordance with the relevant guidelines and regulations.The antibody staining intensity was scored as weak (score 1), medium (score 2) or high (score 3). The extent of staining was scored as the number of cells stained: < 25% (score 1), 25–75% (score 2) and > 75% (score 3). The final score was calculated as the product of the intensity- and the extent-scores. Final score categories were negative (score 0), medium expression (1–4), and high expression (5–9). All scores were assessed in consensus by two observers, following an independent evaluation. Fisher’s exact probability test (two-sided) was used for all statistical analyses, with P < 0.05 considered to indicate statistical significance.

## Supplementary Information

Supplementary Information 1.

Supplementary Table S1.

Supplementary Table S2.

Supplementary Table S3.

Supplementary Table S4.

## Data Availability

All raw mass spectrometry proteomics data files, MaxQuant output files and MaxQuant software used for the search are available from the ProteomeXchange Consortium via the PRIDE partner repository with the dataset identifier PXD020194.
