# Supplementary material for: Proteomic comparison between different tissue preservation methods for identification of promising biomarkers of urothelial bladder cancer
Source: Sci Rep. 2021 Apr 7;11:7595. doi: 10.1038/s41598-021-87003-6 (PMC8027873; doi:10.1038/s41598-021-87003-6)
Supplement: Supplementary file 1 — Supplementary Information 1. [file 41598_2021_87003_MOESM1_ESM.pdf]

Supplementary Information for

Proteomic comparison between different tissue preservation methods for identification of promising biomarkers of urothelial bladder cancer

Alberto Valdés[1,2]*, Athanasios Bitzios[1]*, Eszter Kassa[1], Ganna Shevchenko[1], Alexander Falk[1], Per-Uno Malmström[3], Anca Dragomir[4], Ulrika Segersten[3], Sara Bergström Lind[1]**

[1] Department of Chemistry-BMC, Analytical Chemistry, Uppsala University, Box 599, 75124 Uppsala, Sweden.

[2] Laboratory of Foodomics, Institute of Food Science Research, CIAL, CSIC, Nicolás Cabrera 9, 28049 Madrid, Spain.

[3] Department of Surgical Science, Urology, Uppsala University, Akademiska sjukhuset, 751 85 Uppsala.

[4] Department of Immunology, Genetics and Pathology, Uppsala University, and Department of Pathology, Uppsala University Hospital, 751 85 Uppsala.

* Shared first authorship.

** Corresponding author. Associate Prof. Sara Bergström Lind, Department of Chemistry-BMC, Analytical Chemistry, Uppsala University, Box 599, 751 24 Uppsala, Sweden. Email: sara.lind@uadm.uu.se, telephone: +46 18 471 5345.

Table of contents

Supplementary Figure S1. Overlap of proteins identified in FFPE and OCT samples from nine different patients. Patients 11, 2, 7 and 9 were diagnosed with stage T2/T3; patients 12, 13, 14, 19 and 20 were diagnosed with stage Ta/T1.

Supplementary Figure S2. Scatterplots of the log2 transformed LFQ intensities of proteins commonly quantified in stage T2/T3 tumors from the same patient and preserved by FFPE and OCT methods. The correlations are presented by Pearson's correlation coefficients (r).

Supplementary Figure S3. Scatterplots of the log2 transformed LFQ intensities of proteins commonly quantified in stage Ta/T1 tumors from the same patient and preserved by FFPE and OCT methods. The correlations are presented by Pearson's correlation coefficients (r).

Supplementary Figure S4. PLS-DA score plots (**A** and **B**) and loading plots (**C** and **D**) of data from proteins commonly found among the two tumor stages samples (T2/T3 and Ta/T1) preserved by FFPE (**A** and **C**) and OCT (**B** and **D**).

Supplementary Figure S5. Volcano plot of differentially expressed proteins in FFPE (**A**) and OCT (**B**) preserved samples (T2/T3 vs Ta/T1). Log2 Fold Change threshold: ≥1 or ≤-1; - log p-value threshold: 1.30.

Supplementary Table S1. MaxQuant results for protein identification and quantification.

Supplementary Table S2. List of unique proteins in FFPE and OCT preserved samples.

Supplementary Table S3. Lists of Reactome and Kegg pathways, and Gene Ontology (GO) terms significantly enriched in OCT unique proteins (from Supplementary Table S2) after STRING v11 web-based software analysis.

Supplementary Table S4. Lists of proteins, component contribution, variable importance in the projection (VIP), coefficient values and importance in the separation between the two tumor stages samples preserved by FFPE and OCT after PLS-DA analysis.

Supplementary Table S5. Differentially expressed proteins in FFPE preserved samples (T2/T3 vs Ta/T1).

Supplementary Table S6. Differentially expressed proteins in OCT preserved samples (T2/T3 vs Ta/T1).

Supplementary Table S7. Log2 transformed LFQ intensity of unique proteins in FFPE preserved samples (those quantified in 5 or 6 FFPE samples and in none OCT samples).

Supplementary Table S8. Log2 transformed LFQ intensity of unique proteins in OCT preserved samples (those quantified in 4 or 5 OCT samples and in none FFPE samples).

Supplementary Table S9. Comparison between differentially expressed proteins in FFPE preserved samples (T2/T3 vs Ta/T1) and RNA data (MIBC vs NMIBC) from GSE32894 and GSE83586 data sets (Sjödahl et al., 2012; Sjödahl et al., 2017).

Supplementary Table S10. Comparison between differentially expressed proteins in OCT preserved samples (T2/T3 vs Ta/T1) and RNA data (MIBC vs NMIBC) from GSE32894 and GSE83586 data sets (Sjödahl et al., 2012; Sjödahl et al., 2017).

Supplementary Table S11. Commonly differentially expressed proteins found in FFPE or OCT preserved samples (T2/T3 vs Ta/T1) in the present and in our previous study (Holfeld et al., 2018). BGN and DPYSL2 were found as unique proteins in T2/T3 samples in the previous study.

Supplementary Figure S1.

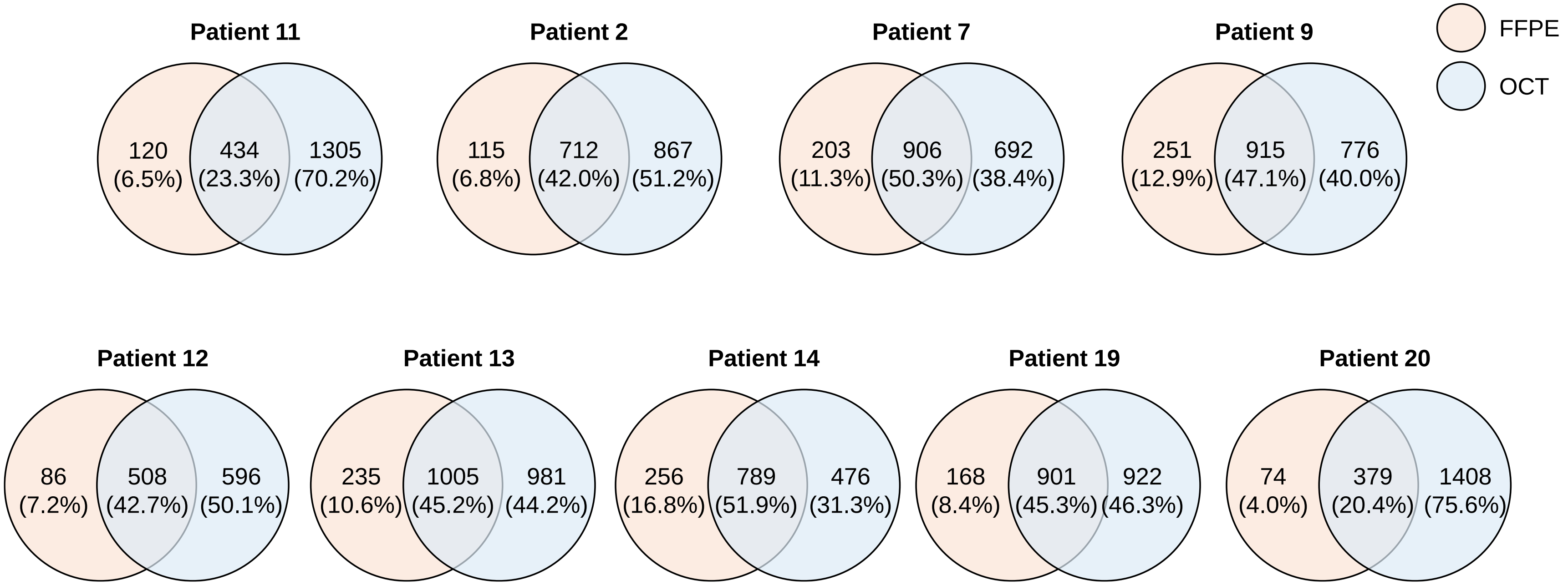

Supplementary Figure S2.

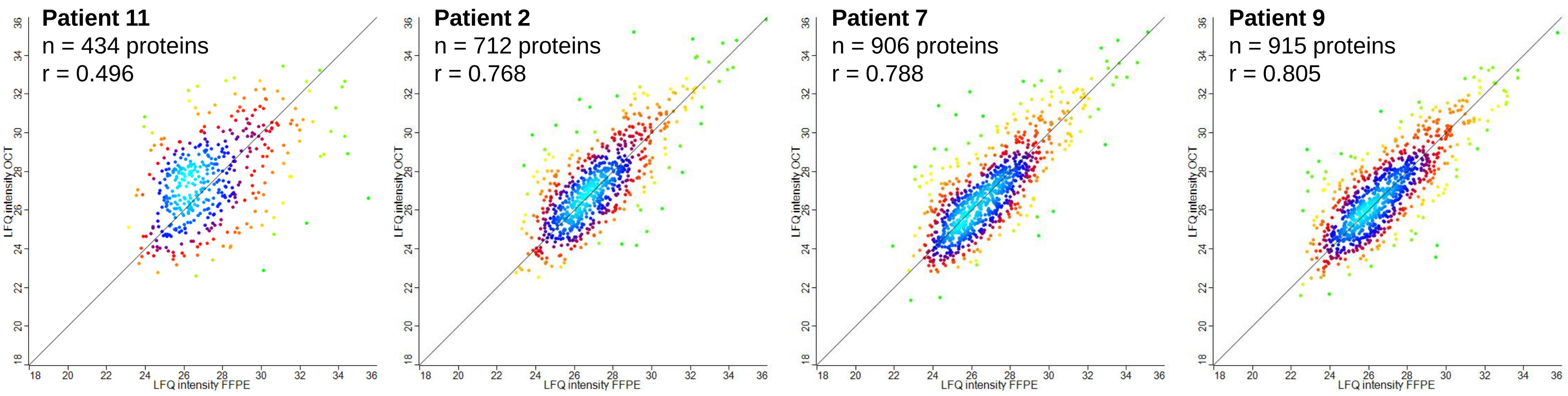

Supplementary Figure S3.

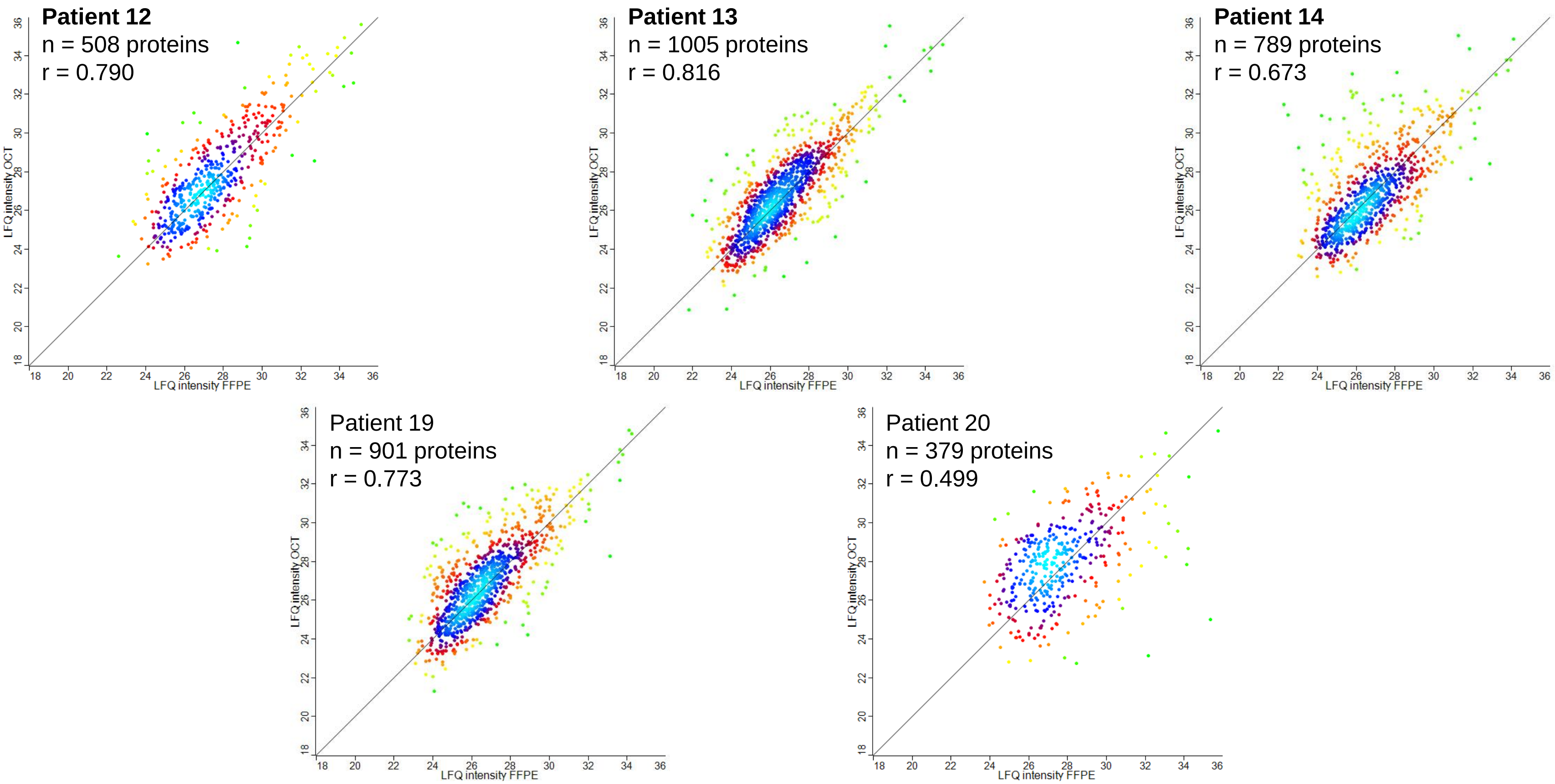

Supplementary Figure S4.

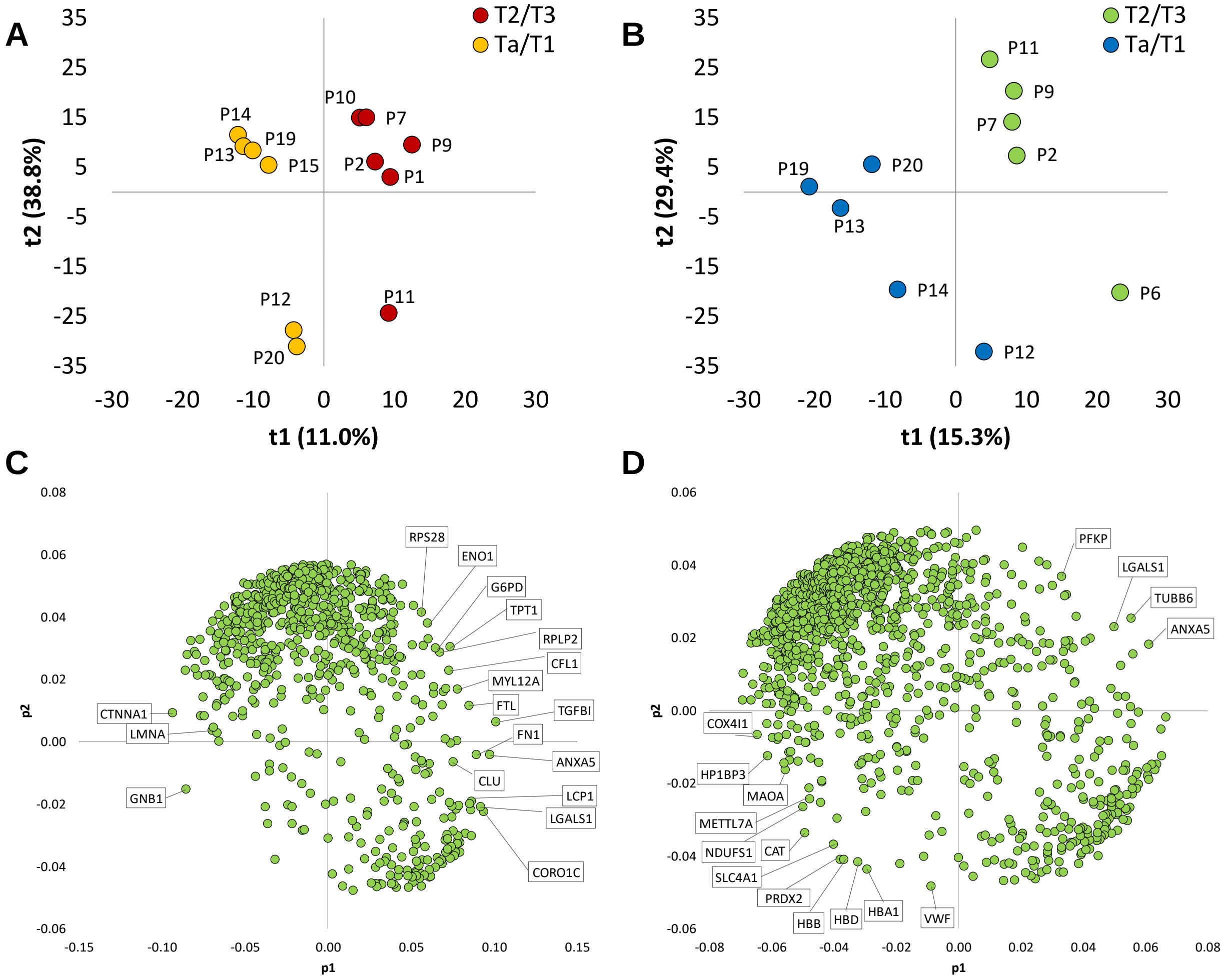

Supplementary Figure S5.

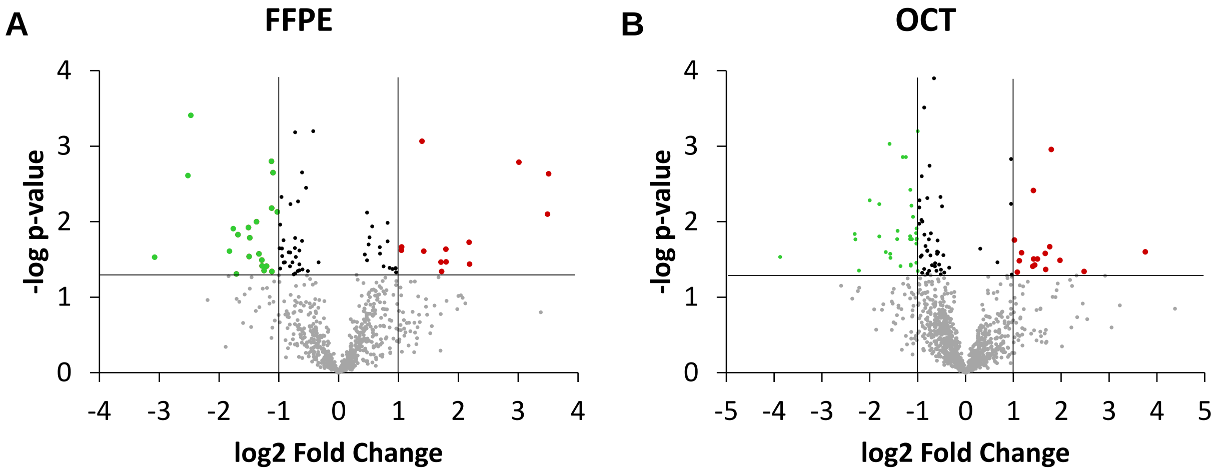

Supplementary Table S5.

Protein ID	Protein name	Gene name	Muscle-Invasive – T2/T3	Nonmuscle-Invasive – Ta/T1	Log2 Fold Change	P-value
Q15582	Transforming growth factor-beta-induced protein ig-h3	TGFBI	27.81	24.30	3.51	0.002
P02751	Fibronectin	FN1	31.76	28.27	3.49	0.008
P02792	Ferritin light chain	FTL	29.31	26.29	3.01	0.002
P16070	CD44 antigen	CD44	27.65	25.45	2.19	0.037
P09382	Galectin-1	LGALS1	29.92	27.73	2.18	0.019
P11413	Glucose-6-phosphate 1-dehydrogenase	G6PD	26.60	24.80	1.80	0.034
P10909	Clusterin	CLU	27.57	25.77	1.79	0.023
P19971	Thymidine phosphorylase	TYMP	27.95	26.22	1.73	0.046
Q13347	Eukaryotic translation initiation factor 3 subunit I	EIF3I	25.26	23.54	1.71	0.034
P13796	Plastin-2	LCP1	29.30	27.88	1.43	0.025
P08758	Annexin A5	ANXA5	29.69	28.30	1.39	0.001
P13693	Translationally-controlled tumor protein	TPT1	27.18	26.13	1.05	0.022
Q16555	Dihydropyrimidinase-related protein 2	DPYSL2	27.59	26.54	1.05	0.024
Q12905	Interleukin enhancer-binding factor 2	ILF2	26.12	27.14	-1.02	0.007
P21964	Catechol O-methyltransferase	COMT	25.17	26.26	-1.09	0.002
P07195	L-lactate dehydrogenase B chain	LDHB	26.74	27.86	-1.11	0.046
O43143	Pre-mRNA-splicing factor ATP-dependent RNA helicase	DHX15	26.07	27.19	-1.12	0.007
Q96PK6	RNA-binding protein 14	RBM14	27.30	28.43	-1.12	0.002
P49411	Elongation factor Tu, mitocondrial	TUFM	26.79	27.99	-1.20	0.039
Q96IU4	Alpha/beta hydrolase domain-containing protein 14B	ABHD14B	25.51	26.76	-1.25	0.045
Q9H0D6	5-3 exoribonuclease 2	XRN2	24.59	25.87	-1.28	0.039
P07814	Bifunctional glutamate/proline--tRNA ligase	EPRS	24.04	25.32	-1.28	0.032
P51659	Peroxisomal multifunctional enzyme type 2	HSD17B4	26.02	27.35	-1.33	0.027
P51532	Transcription activator BRG1	SMARCA4	23.81	25.18	-1.37	0.010
P16615	Sarcoplasmic/endoplasmic reticulum calcium ATPase 2	ATP2A2	25.25	26.73	-1.48	0.016
P17844	Probable ATP-dependent RNA helicase DDX5	DDX5	26.34	27.83	-1.49	0.029
Q9P035	Very-long-chain (3R)-3-hydroxyacyl-CoA dehydratase 3	HACD3	24.20	25.71	-1.50	0.012
P26447	Protein S100-A4	S100A4	27.67	29.35	-1.68	0.015
P09467	Fructose-1,6-bisphosphatase 1	FBP1	25.90	27.61	-1.71	0.049
P78527	DNA-dependent protein kinase catalytic subunit	PRKDC	27.70	29.46	-1.76	0.012
P35221	Catenin alpha-1	CTNNA1	23.98	25.80	-1.82	0.025
P26640	Valine--tRNA ligase	VARS	24.30	26.77	-2.47	0.000
Q04828	Aldo-keto reductase family 1 member C1	AKR1C1	27.50	30.02	-2.52	0.002
Q14980	Nuclear mitotic apparatus protein 1	NUMA1	24.70	27.78	-3.07	0.030

Supplementary Table S6.

Protein ID	Protein name	Gene name	Muscle-Invasive – T2/T3	Nonmuscle-Invasive – Ta/T1	Log2 Fold Change	P-value
P15924	Desmoplakin	DSP	29.83	26.07	3.76	0.025
P04083	Annexin A1	ANXA1	31.07	28.59	2.48	0.046
Q9ULV4	Coronin-1C	CORO1C	27.96	25.98	1.98	0.033
Q9BUF5	Tubulin beta-6 chain	TUBB6	26.04	24.25	1.79	0.001
Q9UGI8	Testin	TES	26.87	25.11	1.76	0.021
P31947	14-3-3 protein sigma	SFN	30.03	28.36	1.67	0.043
P12814	Alpha-actinin-1	ACTN1	30.93	29.26	1.67	0.027
Q9NR30	Nucleolar RNA helicase 2	DDX21	25.17	23.66	1.50	0.031
O15427	Monocarboxylate transporter 4	SLC16A3	24.53	23.08	1.45	0.038
P21810	Biglycan	BGN	29.02	27.59	1.42	0.031
P09382	Galectin-1	LGALS1	29.92	28.50	1.42	0.004
P13797	Plastin-3	PLS3	27.48	26.07	1.41	0.039
Q15942	Zyxin	ZYX	25.34	24.16	1.17	0.026
P36871	Phosphoglucomutase-1	PGM1	26.04	24.92	1.12	0.033
P35237	Serpin B6	SERPINB6	25.39	24.31	1.09	0.047
Q01813	ATP-dependent 6-phosphofructokinase, platelet type	PFKP	27.75	26.73	1.03	0.018
Q9UJS0	Calcium-binding mitochondrial carrier protein Aralar2	SLC25A13	24.57	25.57	-1.00	0.001
Q99878	Histone H2A type 1-J	HIST1H2A	32.16	33.16	-1.00	0.045
P31930	Cytochrome b-c1 complex subunit 1, mitochondrial	UQCRC1	26.35	27.37	-1.02	0.017
P10606	Cytochrome c oxidase subunit 5B, mitochondrial	COX5B	25.28	26.31	-1.02	0.012
P39687	Acidic leucine-rich nuclear phosphoprotein 32 family	ANP32A	26.96	27.98	-1.03	0.017
P49411	Elongation factor Tu, mitochondrial	TUFM	27.53	28.57	-1.03	0.035
O94776	Metastasis-associated protein MTA2	MTA2	24.52	25.56	-1.03	0.019
Q92878	DNA repair protein RAD50	RAD50	24.74	25.77	-1.04	0.014
O94906	Pre-mRNA-processing factor 6	PRPF6	23.41	24.51	-1.10	0.009
Q99873	Protein arginine N-methyltransferase 1	PRMT1	25.97	27.09	-1.12	0.017
P28331	NADH-ubiquinone oxidoreductase 75 kDa subunit,	NDUFS1	24.84	25.97	-1.13	0.006
Q9UHQ9	NADH-cytochrome b5 reductase 1	CYB5R1	24.79	25.94	-1.15	0.038
P48735	Isocitrate dehydrogenase [NADP], mitochondrial	IDH2	28.15	29.30	-1.15	0.037
Q14839	Chromodomain-helicase-DNA-binding protein 4	CHD4	24.75	25.91	-1.16	0.004
P22695	Cytochrome b-c1 complex subunit 2, mitochondrial	UQCRC2	26.01	27.16	-1.16	0.017
Q8TAQ2	SWI/SNF complex subunit SMARCC2	SMARCC2	24.48	25.64	-1.16	0.016
P53007	Tricarboxylate transport protein, mitochondrial	SLC25A1	24.96	26.21	-1.25	0.001
P32119	Peroxiredoxin-2	PRDX2	28.46	29.77	-1.32	0.001
P11310	Medium-chain specific acyl-CoA dehydrogenase,	ACADM	24.47	25.84	-1.36	0.039
Q5SSJ5	Heterochromatin protein 1-binding protein 3	HP1BP3	26.31	27.74	-1.42	0.013
P38117	Electron transfer flavoprotein subunit beta	ETFB	25.58	27.01	-1.43	0.017
Q96I99	Succinyl-CoA ligase [GDP-forming] subunit beta,	SUCLG2	23.95	25.52	-1.57	0.030
P30084	Enoyl-CoA hydratase, mitochondrial	ECHS1	25.64	27.22	-1.58	0.027
P04040	Catalase	CAT	25.44	27.03	-1.59	0.001
Q9H8H3	Methyltransferase-like protein 7A	METTL7A	24.60	26.27	-1.67	0.025
P69905	Hemoglobin subunit alpha	HBA1	32.75	34.56	-1.81	0.016
Q92900	Regulator of nonsense transcripts 1	UPF1	24.51	26.32	-1.81	0.006
P68871	Hemoglobin subunit beta;LVV-hemorphin-7;Spinorphin	HBB	33.74	35.74	-2.00	0.005
P29373	Cellular retinoic acid-binding protein 2	CRABP2	25.15	27.38	-2.23	0.044
Q8TE77	Protein phosphatase Slingshot homolog 3	SSH3	24.14	26.45	-2.31	0.017
P02042	Hemoglobin subunit delta	HBD	28.52	30.84	-2.32	0.015
P02730	Band 3 anion transport protein	SLC4A1	24.06	27.93	-3.88	0.029

Supplementary Table S7.

Protein ID	Protein name	Gene name	Muscle-Invasive – T2/T3 (number of samples)	Nonmuscle-Invasive – Ta/T1 (number of samples)
P54868	Hydroxymethylglutaryl-CoA synthase, mitochondrial	HMGCS2	-	29.04 (5)
P22234	Multifunctional protein ADE2	PAICS	25.31 (5)	-

Supplementary Table S8.

Protein ID	Protein name	Gene name	Muscle-Invasive – T2/T3 (number of samples)	Nonmuscle-Invasive – Ta/T1 (number of samples)
P54868	Hydroxymethylglutaryl-CoA synthase, mitochondrial	HMGCS2	-	28.28 (5)
Q16762	Thiosulfate sulfurtransferase	TST	-	24.51 (5)
P04279	Semenogelin-1	SEMG1	24.16 (4)	-
P04839	Cytochrome b-245 heavy chain	CYBB	24.81 (4)	-
P27144	Adenylate kinase 4, mitochondrial	AK4	25.09 (4)	-
P50479	PDZ and LIM domain protein 4	PDLIM4	26.05 (4)	-
Q15113	Procollagen C-endopeptidase enhancer 1	PCOLCE	24.57 (4)	-
Q8IVF2	Protein AHNAK2	AHNAK2	25.73 (4)	-
Q96S97	Myeloid-associated differentiation marker	MYADM	25.15 (4)	-

Supplementary Table S9.

			Protein data from FFPE (T2/T3 vs Ta/T1)		RNA data from GSE32894 (MIBC vs NMIBC)		RNA data from GSE83586 (MIBC vs NMIBC)	
**Protein ID**	**Protein name**	**Gene name**	**Log2 Fold Change**	**P-value**	**Log2 Fold Change**	**adj P-value**	**Log2 Fold Change**	**adj P-value**
Q15582	Transforming growth factor-beta-induced protein ig-h3	TGFBI	3.51	0.0023	1.31	2.11E-15	0.66	1.66E-03
P02751	Fibronectin	FN1	3.49	0.0080	0.21	3.15E-06	1.24	4.57E-07
P02792	Ferritin light chain	FTL	3.01	0.0016	0.16	3.47E-02	0.16	1.69E-01
P16070	CD44 antigen	CD44	2.19	0.0367	0.33	2.37E-03	0.46	4.57E-02
P09382	Galectin-1	LGALS1	2.18	0.0188	0.99	8.17E-11	0.59	3.50E-04
P11413	Glucose-6-phosphate 1-dehydrogenase	G6PD	1.80	0.0341	0.48	1.15E-07	0.07	7.37E-01
P10909	Clusterin	CLU	1.79	0.0232	0.00	9.57E-01	-0.05	9.12E-01
P19971	Thymidine phosphorylase	TYMP	1.73	0.0459	0.67	3.48E-12	0.31	3.69E-02
Q13347	Eukaryotic translation initiation factor 3 subunit I	EIF3I	1.71	0.0344	-0.06	2.72E-01	0.00	9.94E-01
P13796	Plastin-2	LCP1	1.43	0.0248	1.08	9.08E-10	0.25	3.20E-01
P08758	Annexin A5	ANXA5	1.39	0.0009	0.69	1.01E-08	0.57	9.33E-05
P13693	Translationally-controlled tumor protein	TPT1	1.05	0.0218	-0.17	8.14E-02	-0.04	8.98E-01
Q16555	Dihydropyrimidinase-related protein 2	DPYSL2	1.05	0.0240	0.78	6.77E-10	0.11	4.78E-01
Q12905	Interleukin enhancer-binding factor 2	ILF2	-1.02	0.0075	0.07	3.44E-01	-0.07	6.17E-01
P21964	Catechol O-methyltransferase	COMT	-1.09	0.0023	-0.44	1.02E-07	-0.20	5.85E-02
P07195	L-lactate dehydrogenase B chain	LDHB	-1.11	0.0458	-0.48	2.38E-07	-0.18	3.94E-01
O43143	Pre-mRNA-splicing factor ATP-dependent RNA helicase	DHX15	-1.12	0.0066	-0.04	5.11E-01	-0.17	1.39E-01
Q96PK6	RNA-binding protein 14	RBM14	-1.12	0.0016	0.08	6.26E-02	0.05	6.43E-01
P49411	Elongation factor Tu, mitochondrial	TUFM	-1.20	0.0391	-0.14	4.46E-02	-0.13	1.18E-01
Q96IU4	Alpha/beta hydrolase domain-containing protein 14B	ABHD14B	-1.25	0.0445	-0.38	2.19E-07	-0.07	4.58E-01
Q9H0D6	5-3 exoribonuclease 2	XRN2	-1.28	0.0386	-0.05	4.93E-01	-0.05	7.54E-01
P07814	Bifunctional glutamate/proline--tRNA ligase	EPRS	-1.28	0.0323	0.01	9.01E-01	0.00	9.90E-01
P51659	Peroxisomal multifunctional enzyme type 2	HSD17B4	-1.33	0.0268	-0.40	4.06E-09	-0.28	1.26E-02
P51532	Transcription activator BRG1	SMARCA4	-1.37	0.0101	-0.03	6.53E-01	-0.08	4.56E-01
P16615	Sarcoplasmic/endoplasmic reticulum calcium ATPase 2	ATP2A2	-1.48	0.0164	0.11	2.33E-01	0.05	6.91E-01
P17844	Probable ATP-dependent RNA helicase DDX5	DDX5	-1.49	0.0290	0.02	8.45E-01	-0.13	1.78E-01
Q9P035	Very-long-chain (3R)-3-hydroxyacyl-CoA dehydratase 3	HACD3	-1.50	0.0120	-0.43	2.13E-06	-0.13	5.06E-01
P26447	Protein S100-A4	S100A4	-1.68	0.0149	-0.73	1.73E-04	-0.62	9.69E-03
P09467	Fructose-1,6-bisphosphatase 1	FBP1	-1.71	0.0494	-1.06	1.88E-10	-0.86	4.16E-05
P78527	DNA-dependent protein kinase catalytic subunit	PRKDC	-1.76	0.0124	-0.05	4.07E-01	0.02	9.37E-01
P35221	Catenin alpha-1	CTNNA1	-1.82	0.0248	-0.28	2.19E-06	-0.10	2.74E-01
P26640	Valine--tRNA ligase	VARS	-2.47	0.0004	0.00	9.56E-01	0.02	8.81E-01
Q04828	Aldo-keto reductase family 1 member C1	AKR1C1	-2.52	0.0025	-	-	-0.32	4.70E-01
Q14980	Nuclear mitotic apparatus protein 1	NUMA1	-3.07	0.0296	-0.28	1.94E-04	-0.02	9.06E-01
P54868	Hydroxymethylglutaryl-CoA synthase, mitochondrial	HMGCS2	Unique in Ta/T1		-2.35	2.11E-14	-1.03	2.24E-03
P22234	Multifunctional protein	PAICS	Unique in T2/T3		0.01	9.40E-01	0.01	9.71E-01

Supplementary Table S10.

Protein ID	Protein name	Gene name	Protein data from OCT (T2/T3 vs Ta/T1)		RNA data from GSE32894 (MIBC vs NMIBC)		RNA data from GSE83586 (MIBC vs NMIBC)	
			Log2 Fold Change	P-value	Log2 Fold Change	adj P-value	Log2 Fold Change	adj P-value
P15924	Desmoplakin	DSP	3.76	0.0252	0.12	4.93E-02	0.56	3.90E-02
P04083	Annexin A1	ANXA1	2.48	0.0457	0.92	2.78E-08	0.66	1.80E-02
Q9ULV4	Coronin-1C	CORO1C	1.98	0.0328	0.63	9.22E-15	0.49	5.02E-05
Q9BUF5	Tubulin beta-6 chain	TUBB6	1.79	0.0011	0.63	5.55E-10	0.28	1.61E-02
Q9UGI8	Testin	TES	1.76	0.0215	-0.03	6.77E-01	0.07	7.47E-01
P31947	14-3-3 protein sigma	SFN	1.67	0.0432	0.30	4.32E-02	0.52	3.09E-02
P12814	Alpha-actinin-1	ACTN1	1.67	0.0265	0.85	2.05E-12	0.60	1.59E-05
Q9NR30	Nucleolar RNA helicase 2	DDX21	1.50	0.0314	0.15	1.44E-01	0.11	4.36E-01
O15427	Monocarboxylate transporter 4	SLC16A3	1.45	0.0377	0.52	6.94E-06	0.07	6.36E-01
P21810	Biglycan	BGN	1.42	0.0313	0.69	1.13E-06	0.52	7.29E-03
P09382	Galectin-1	LGALS1	1.42	0.0039	0.99	8.17E-11	0.59	3.50E-04
P13797	Plastin-3	PLS3	1.41	0.0391	0.29	1.35E-04	0.17	4.43E-01
Q15942	Zyxin	ZYX	1.17	0.0260	0.39	4.31E-08	0.21	1.16E-01
P36871	Phosphoglucomutase-1	PGM1	1.12	0.0331	0.14	2.87E-01	0.28	2.54E-02
P35237	Serpin B6	SERPINB6	1.09	0.0467	0.10	1.65E-01	0.05	6.76E-01
Q01813	ATP-dependent 6-phosphofructokinase, platelet	PFKP	1.03	0.0176	-0.03	3.77E-01	0.22	2.16E-01
Q9UJS0	Calcium-binding mitochondrial carrier protein	SLC25A13	-1.00	0.0006	0.07	3.57E-01	0.03	8.44E-01
Q99878	Histone H2A type 1-J	HIST1H2AJ	-1.00	0.0450	-	-	0.21	3.77E-01
P31930	Cytochrome b-c1 complex subunit 1, mitochondrial	UQCRC1	-1.02	0.0168	-0.08	2.18E-01	0.00	9.90E-01
P10606	Cytochrome c oxidase subunit 5B, mitochondrial	COX5B	-1.02	0.0122	-0.21	2.54E-04	-0.07	4.97E-01
P39687	Acidic leucine-rich nuclear phosphoprotein 32 family	ANP32A	-1.03	0.0171	-0.10	2.65E-01	0.01	9.66E-01
P49411	Elongation factor Tu, mitochondrial	TUFM	-1.03	0.0351	-0.14	4.46E-02	-0.13	1.18E-01
O94776	Metastasis-associated protein MTA2	MTA2	-1.03	0.0194	0.05	3.67E-01	0.03	8.24E-01
Q92878	DNA repair protein RAD50	RAD50	-1.04	0.0141			-0.19	1.28E-01
O94906	Pre-mRNA-processing factor 6	PRPF6	-1.10	0.0086	-0.14	2.63E-03	-0.03	8.08E-01
Q99873	Protein arginine N-methyltransferase 1	PRMT1	-1.12	0.0170	-0.07	5.15E-01	-0.03	8.24E-01
P28331	NADH-ubiquinone oxidoreductase 75 kDa subunit,	NDUFS1	-1.13	0.0061	-0.14	5.16E-04	-0.09	4.63E-01
Q9UHQ9	NADH-cytochrome b5 reductase 1	CYB5R1	-1.15	0.0381	-0.39	1.21E-03	-	-
P48735	Isocitrate dehydrogenase [NADP], mitochondrial	IDH2	-1.15	0.0370	0.12	2.08E-01	0.09	5.08E-01
Q14839	Chromodomain-helicase-DNA-binding protein 4	CHD4	-1.16	0.0038	-0.03	6.18E-01	-0.02	8.82E-01
P22695	Cytochrome b-c1 complex subunit 2, mitochondrial	UQCRC2	-1.16	0.0170	-0.42	1.23E-09	-0.18	3.72E-02
Q8TAQ2	SWI/SNF complex subunit SMARCC2	SMARCC2	-1.16	0.0155	-0.20	1.87E-03	-0.17	1.50E-02
P53007	Tricarboxylate transport protein, mitochondrial	SLC25A1	-1.25	0.0014	-0.16	4.07E-02	-0.10	4.45E-01
P32119	Peroxiredoxin-2	PRDX2	-1.32	0.0014	-	-	-0.11	2.12E-01
P11310	Medium-chain specific acyl-CoA dehydrogenase,	ACADM	-1.36	0.0387	-0.34	5.42E-04	-0.22	1.38E-01
Q5SSJ5	Heterochromatin protein 1-binding protein 3	HP1BP3	-1.42	0.0132	0.06	2.21E-01	0.02	9.12E-01

P38117	Electron transfer flavoprotein subunit beta	ETFB	-1.43	0.0170	-0.31	1.40E-03	0.03	8.55E-01
Q96I99	Succinyl-CoA ligase [GDP-forming] subunit beta,	SUCLG2	-1.57	0.0301	-0.42	2.38E-08	-0.18	1.51E-01
P30084	Enoyl-CoA hydratase, mitochondrial	ECHS1	-1.58	0.0267	-0.32	3.92E-05	-0.14	2.49E-01
P04040	Catalase	CAT	-1.59	0.0009	-0.53	1.07E-07	-0.27	4.02E-02
Q9H8H3	Methyltransferase-like protein 7A	METTL7A	-1.67	0.0252	-0.74	1.92E-08	-0.25	2.61E-01
P69905	Hemoglobin subunit alpha	HBA1	-1.81	0.0156	-	-	-	-
Q92900	Regulator of nonsense transcripts 1	UPF1	-1.81	0.0058	-0.03	7.29E-01	-0.05	5.00E-01
P68871	Hemoglobin subunit beta;LVV-hemorphin-	HBB	-2.00	0.0052	0.12	6.45E-01	-0.38	1.36E-01
P29373	Cellular retinoic acid-binding protein 2	CRABP2	-2.23	0.0443	0.51	1.76E-02	-0.18	7.08E-01
Q8TE77	Protein phosphatase Slingshot homolog 3	SSH3	-2.31	0.0171	-1.08	2.88E-16	-0.32	4.28E-02
P02042	Hemoglobin subunit delta	HBD	-2.32	0.0146	0.00	9.96E-01	0.01	9.30E-01
P02730	Band 3 anion transport protein	SLC4A1	-3.88	0.0292	-	-	0.04	6.55E-01
P54868	Hydroxymethylglutaryl-CoA synthase, mitochondrial	HMGCS2	Unique in Ta/T1		-2.35	2.11E-14	-1.03	2.24E-03
Q16762	Thiosulfate sulfurtransferase	TST	Unique in Ta/T1		-0.46	2.46E-06	-0.09	5.91E-01
P04279	Semenogelin-1;Alpha-inhibin-92;Alpha-inhibin-	SEMG1	Unique in T2/T3		0.12	5.99E-02	0.04	7.54E-01
P04839	Cytochrome b-245 heavy chain	CYBB	Unique in T2/T3		0.76	6.86E-11	0.39	5.96E-02
P27144	Adenylate kinase 4, mitochondrial	AK4	Unique in T2/T3		0.23	1.15E-02	0.23	1.88E-01
P50479	PDZ and LIM domain protein 4	PDLIM4	Unique in T2/T3		0.17	5.74E-06	0.27	2.19E-02
Q15113	Procollagen C-endopeptidase enhancer 1	PCOLCE	Unique in T2/T3		0.63	5.57E-08	0.31	7.06E-02
Q8IVF2	Protein AHNAK2	AHNAK2	Unique in T2/T3		1.18	1.04E-09	0.85	1.38E-04
Q96S97	Myeloid-associated differentiation marker	MYADM	Unique in T2/T3		0.56	1.20E-07	0.36	2.00E-02

Supplementary Table S11.

			Previous Study			Present Study		
**Protein ID**	**Protein name**	**Gene name**	**Log2 Fold Change**	**P-value**	**Preservation method**	**Log2 Fold Change**	**P-value**	**Preservation method**
P12814	Alpha-actinin-1	ACTN1	2.58	0.031	FFPE	1.67	0.027	OCT
P21810	Biglycan	BGN	**T2/T3**	-	FFPE	1.42	0.031	OCT
P13796	Plastin-2	LCP1	1.18	0.008	FPPE	1.43	0.025	FFPE
P08758	Annexin A5	ANXA5	1.03	0.001	FFPE	1.39	0.001	FFPE
Q16555	Dihydropyrimidinase-related protein 2	DPYSL2	**T2/T3**	-	FFPE	1.05	0.024	FFPE
P31930	Cytochrome b-c1 complex subunit 1, mitochondrial	UQCRC1	-1.07	0.022	FFPE	-1.02	0.017	OCT
P51659	Peroxisomal multifunctional enzyme type 2	HSD17B4	-1.07	0.022	FFPE	-1.33	0.027	FFPE
P26640	Valine--tRNA ligase	VARS	-1.54	0.007	FFPE	-2.47	0.001	FFPE
